# Molecular Phylogeny and Biogeography of the Hawaiian Craneflies *Dicranomyia* (Diptera: Limoniidae)

**DOI:** 10.1371/journal.pone.0073019

**Published:** 2013-09-13

**Authors:** Kari Roesch Goodman, Patrick O'Grady

**Affiliations:** 1 Department of Environmental Science, Policy and Management, University of California, Berkeley, California, United States of America; 2 Bishop Museum, Honolulu, Hawaii, United States of America; CNRS, France

## Abstract

The Hawaiian Diptera offer an opportunity to compare patterns of diversification across large and small endemic radiations with varying species richness and levels of single island endemism. The craneflies (Limoniidae: *Dicranomyia*) represent a small radiation of 13 described species that have diversified within the Hawaiian Islands. We used Bayesian and maximum likelihood approaches to generate a molecular phylogeny of the Hawaiian *Dicranomyia* using a combination of nuclear and mitochondrial loci, estimated divergence times and reconstructed ancestral ranges. Divergence time estimation and ancestral range reconstruction suggest that the colonization that led to most of the diversity within the craneflies arrived prior to the formation of Kauai and demonstrates that the two major clades within that radiation contrast sharply in their patterns of diversification.

## Introduction

The Hawaiian Islands contain a large number of evolutionary radiations across a diversity of plant and animal groups (e.g. [1], [2], [3], [4], [5], [6], [7]), making it an “unparalleled scientific laboratory for studying processes of evolution” [8]. Arthropods contain some of the most spectacular of these radiations, with some lineages numbering in the dozens or even hundreds of species and single island endemism rates approaching 99% [9]. These radiations, each arising from independent colonization events, offer an opportunity to compare patterns of diversification across lineages with different numbers of species, ecological roles, and ages of colonization. Diptera are particularly interesting with 24 families containing endemic taxa in Hawaii, eight of which contain radiations with more than ten species (Table 1).

**Table 1:** Hawaiian Diptera lineages with more than 10 endemic species.

Family	Genus/complex	# of species*
Limoniidae	*Dicranomyia* [*^25^*]	13
Ephydridae	*Scatella* [*^25^*]	18
Calliphoridae	*Dyscritiomyia* [*^25^*]	25
Tephritidae	*Trupanea* [*^25]^*, [*^78^*]	25
Pipunculidae	*Cephalops* [*^25^*]	26
Dolichopodidae	Eurynogaster Complex[*^79^*]	70+
Muscidae	*Lispocephala* [*^25^*]	102
Drosophilidae	*Scaptomyza* [*^25^*]	155+
Dolichopodidae	*Campsicnemus* [*^79^*]	242
Drosophilidae	*Drosophila* [*^25^*]	403+

* + indicates undescribed species in the group.

Geological heterogeneity has been proposed as a potential factor driving diversification in Hawaiian lineages and the unique geology of the Hawaiian Islands may contribute to rapid species formation ([10] and references therein). The Hawaiian – Emperor chain is an archipelago formed by the motion of the Pacific plate over a stationary hotspot [11]. This has generated an archipelago that has formed sequentially in the central Pacific Ocean over the past approximately 80 million years [12], [13], [14]. The eight contemporary high Hawaiian Islands (in chronological sequence from oldest to youngest: Niihau, Kauai, Oahu, Molokai, Lanai, Maui, Kahoolawe and Hawaii) that comprise the south-eastern corner of this chain have been forming over the past approximately five million years [14], [15]. Each island is physically isolated from the others by open ocean, although during historical periods of lower sea levels, the islands comprising the Maui Nui complex (Molokai, Lanai, Maui, Kahoolawe) were connected during much of their histories [16]. Each island is further subdivided into habitat patches generated by the distinctive within-island microclimates and the active volcanic and erosional processes that give them their characteristically rugged profiles [17]. These conditions combine to allow for both between and within island diversification of many of the taxa inhabiting them.

The largest endemic Hawaiian lineage is the Drosophilidae [18], [19], [20], a clade estimated to contain over 1000 species [21]. Hawaiian Drosophilidae tend to have small ranges, with approximately 90% of species endemic to a single island and a high number of taxa endemic to single volcanoes [22], [23]. Drosophilidae are also known to exploit a wide range of host plants within high elevation rainforests and have been reared from 34 of the 87 endemic Hawaiian plant genera [14]. Diversification of Hawaiian Drosophilidae may be driven by a combination of geographic and ecological forces, mediated by host plant specialization and microbial community [24]. While Drosophilidae constitute a spectacular example of an adaptive radiation, the Hawaiian Islands are also home to several smaller dipteran radiations [9], [25], [26], each derived from one or more independent colonization events and comprised of species whose geographic ranges, dispersal abilities, and other life history traits provide a contrast to the Drosophilidae.

One smaller radiation of Hawaiian Diptera is the *Dicranomyia* craneflies in the family Limoniidae. Larvae in this family are known to use a wide diversity of habitats, including a spectrum of aquatic habitats such as running and stagnant freshwater, brackish pools, algae and other plant material present in the intertidal zone and the leaves of terrestrial plants [27], [28]. Within the Hawaiian islands, however, very little is known about *Dicranomyia* biology or ecology. Their larvae are aquatic or semi-aquatic [29]. Little else is known about the immature stages in these Hawaiian taxa, though there is at least one leaf-mining species [30], and others have been observed in dripping wet banks, algal growth on rocks in mountain streams, and tree holes or leaf axils filled with water [31]. *Dicranomyia grimshawi* larvae have been observed feeding on the pupae of *D. jacobus* [31], though their primary food source appears to be decaying plant tissue. Adults are typically collected in moist, dark places like shady spots near streams, in dense mountain vegetation and entrances to caves [29]. They are detritivores, feeding on decomposing plant material and associated microbes, although some are known to feed directly on mosses and liverworts [27]. It is possible that, while adults appear to share similar ecological roles in the Hawaiian Islands, larval ecology may be quite distinct among species.

Thirteen *Dicranomyia* species are known from the Hawaiian Islands [29], [32], [33], [34]. The Hawaiian *Dicranomyia* were treated by Hardy [29], with subsequent work by Byers [32], [33], [34]. Beyond the basic alpha taxonomy, there has been very little ecological or evolutionary work done on this group. Seven species are present on all of the current high islands (Kauai, Oahu, Molokai, Maui, Lanai, Hawaii), three are found on more than one high island, and three have distributions restricted to single islands. Although their diversity on Hawaii is small, they belong to the largest genus in the largest and one of the oldest families of Diptera [35], [36]. Craneflies in the family Limoniidae contain 10,541 currently recognized species [37] with fossils that date to the upper Triassic approximately 208 million years ago [38]. *Dicranomyia* is the largest genus within the Limoniidae, containing 1,086 species distributed worldwide. Of the 148 Limoniidae genera, only one other genus is comparably large, containing more than 1,000 species (*Molophilus*) while two additional genera contain more than 500 species (*Gonomyia and Hexatoma*) [37]. Based on a recent study of 88 morphological characters from 104 species within Limoniidae, *Dicranomyia* appears to be a relatively derived genus within the family [39]. Oosterbroek [37] reports 236 *Dicranomyia* species from the Oceanic region, including Australia and New Zealand. The colonization pathway to Hawaii is presently unknown.

Nitta and O'Grady [40] examined four mitochondrial loci from eight of the 13 endemic Hawaiian *Dicranomyia*, and found that species with populations distributed on multiple islands had complex histories that didn't conform to patterns seen in other Hawaiian radiations [41]. Their data also suggested that species in this group may be derived from two separate colonization events into the Hawaiian archipelago, one that led to the majority of Hawaiian diversity, and another represented by a single species, *D. iniquispina*. In this study, we build on previous phylogenetic work [40] by expanding species, geographic, and gene sampling. We reconstruct phylogenetic relationships for this group, estimate divergence dates, and reconstruct ancestral ranges to analyze the biogeographic history of *Dicranomyia* in Hawaii.

## Methods

### Taxonomic sampling

Specimens were collected from sites across the Hawaiian Islands and French Polynesia (Appendix S1 in File S1) by general sweeping in moist areas such as streams and seeps. All material was preserved in 95% ethanol. Collecting permits for public land in Hawaii were issued by the State of Hawaii's Department of Land and Natural Resources and the National Park Service, and permission to collect on private land was granted by Maui Land and Pineapple, East Maui Irrigation, and Parker Ranch. Collections in French Polynesia were made from public land and permits were obtained from the Delegation a la Recherche. No protected species were sampled as a part of this work.

Hardy's [29] key was used to identify Hawaiian material. A number of resources were employed to identify taxa from French Polynesia [42], [43], [44], [45], [46], [47], [48], [49], [50]. Wings and mounted genitalia were preserved as vouchers for all DNA accessions used in this study. Whenever possible, a series of conspecifics from the same collection site and date were also preserved in 95% ethanol. Voucher material has been deposited in the Bernice P. Bishop Museum (Honolulu, HI) and the Essig Museum of Entomology (U.C. Berkeley).

Nine of the 13 species of Hawaiian *Dicranomyia* were sampled for this study. Three of the unsampled species are flightless single mountaintop endemics [32], [33], [34] that are very rare and were not possible to collect. One additional species (*D. nigropolita*) was not collected. All *Dicranomyia* species included in this study are known to have populations on multiple islands, and every effort was made to sample as widely as possible from throughout their known geographic ranges. The ingroup includes geographic representation from as much of the known range of each species as possible. Five outgroup species thought to be closely related to the Hawaiian *Dicranomyia* were included: the congeneric species *D. tahitiensis* from French Polynesia, and three species from within the same subfamily as *Dicranomyia*, Limoniinae: *Geronomyia advena, Libnotes orofenaae* and *Libnotes perkinsi* from Hawaii and French Polynesia. The fifth outgroup taxon was *Styringomyia didyma*, a species in the subfamily Chioneinae (Appendix S1 in File S1).

### DNA extraction, amplification and sequencing

Genomic DNA was extracted from individuals using a Qiagen DNeasy® DNA extraction kit (Qiagen Inc.), following the manufacturer's protocol. Four mitochondrial (COI, COII, ND2, 16S) and two nuclear loci (SNF, CAD) were then amplified and sequenced to estimate phylogenetic relationships within this group (see primer information in Appendix S2 in File S1). PCR reactions were performed using standard master mixes of 25 μL final volumes including: 1.5–3 μL DNA, 2.5 μL of 10X PCR Buffer (BioRad), 0.5 μL of 10 mM dNTPs (New England BioLabs), 1.25–2 μL of each primer (1:9 dilution), 0.75–2 μL of 50 mM MgCl_2_ (BioRad), 0.125 μL of 5 U/μL iTaq® (BioRad) and 14.175–15.62 μL ddH_2_O. Thermal cycling involved either a simple protocol for the mitochondrial genes (described in [40]), a nested reaction for CAD (described in [51]), or a simple protocol for SNF, which began with an initial denaturing step at 95°C for 4 minutes, 30 cycles of 90°C for 30 s, 54°C–58°C for 30 s, 72°C for 60 s and a final extension for 5–10 minutes 72°C. PCR products were purified using *ExoSAP-IT* (USB Corporation, Cleveland, OH) following standard protocols, and the products were sent to the UC Berkeley DNA Sequencing Center for sequencing in both directions on an ABI 3730 capillary sequencer.

### Sequence Editing and Alignment

Contigs were assembled from raw forward and reverse sequence reads and edited using Geneious Pro 5.4.6 (Biomatters). The ClustalW Alignment plugin in Geneious was used to create an aligned data matrix. Alignments for each gene were imported into MacClade 4.08 [52] in order to calculate codon positions using the conceptual translation and comparison to a *Drosophila yakuba* reference sequence. The 16S locus is non-coding and was adjusted manually.

### Phylogenetic Analysis

Analyses were performed on each gene individually and on the combined dataset, using both maximum likelihood (ML) and Bayesian inference (BI) optimality criteria. Individual ML analyses were performed on each gene partition using PhyML [53] in Geneious Pro 5.4.6 (Biomatters) under a general time-reversible (GTR+GAMMA) model [54] with 200 bootstrap replicates. PartitionFinder [55] was used to determine the optimum partitioning scheme and the best fit nucleotide models for each partition for the individual genes and combined data sets, selected using Bayesian Information Criterion (BIC). These partitions and models (Appendix S3 in File S1) were applied to the individual BI analyses, which were run for 5,000,000 generations with 2 independent runs using MrBayes 3.1.2 [56] on CIPRES [57].

The concatenated data set consisted of 45 individuals and 6 loci (3880 bp). The partition and model selection procedure yielded nine partitions for the final analyses, each presented with its best-fit model in Appendix S3 in File S1. ML analysis was performed on the concatenated data set in RAxML 3.7.2 [58] on CIPRES [57] under the GTR+GAMMA model with 1,000 bootstrap replicates and a final search for the best tree. Concatenated BI analyses were performed using MrBayes 3.1.2 [56] on CIPRES [57], with the analysis run for 15,000,000 generations with 4 independent runs each. Stationarity in BI runs was assessed using several complimentary approaches: (1) convergence metrics provided by MrBayes 3.1.2 were checked (Huelsenbeck and Ronquist, 2001) to ensure that the maximum standard deviation of split frequencies of any of the runs was under 0.05 and that the potential scale reduction factor for all parameters approached 1.0, and (2) the log-likelihood values for each run were plotted, the effective sample sizes were checked to ensure there were an adequate number of independent samples, and the posterior distributions of all parameters were examined using Tracer v.1.7.2 [59]. Tracer v.1.7.2 was also used to determine the burn-in phase by assessing each run's plot of log-likelihood values over generations; stationarity was assumed to have been reached when the log likelihood values reached a stable plateau (*i.e.* bounced around a mean rather than rising). Finally, a 50% majority rule consensus tree was created from the resulting post burn-in trees.

### Divergence Time Estimation

Divergence time estimation was performed using a Bayesian relaxed-clock method implemented in Beast 1.7.4 [60] on CIPRES [57]. Molecular clocks can be calibrated using fossils or biogeography, or they can be set using approximations of known molecular rates. While fossils do exist in the genus *Dicranomyia*, they are quite old (∼200 million years) and are very distantly related to the Hawaiian members of this genus. Taxon sampling in this project focused on the taxa endemic to the Hawaiian Islands and was not intended to represent genus-wide diversity within *Dicranomyia*. Therefore, it was inappropriate to apply the known fossils as calibrations in this analysis [61]. Instead, we estimated dates using island calibrations and divergence rates. Because each approach has its own set of limitations (discussed below), in order to fully explore dating parameter space, we performed two calibration-based estimates and two rates-based estimates.

#### Island calibrations

**Figure 1:**
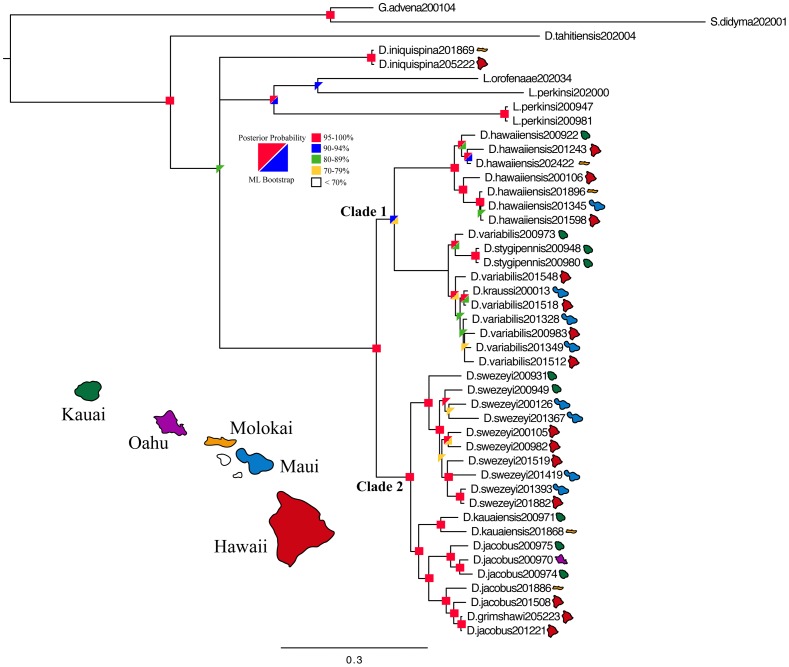
Majority rule consensus tree summarizing Bayesian analysis of *Dicranomyia.* Bayesian posterior probabilities and bootstrap supports from the maximum likelihood analysis are displayed as colored boxes. Islands that each specimen was collected from are shown next to each tip.

The application of island calibrations rests on the assumption that a taxon colonized a new island from an older island after it emerged and thus the age of that biogeographic event can then be used to date the most recent common ancestor (MRCA) of that group. Thus, in order to apply calibrations, pairs of taxa must be used in which one group is restricted to an older island (or islands) and the other group is restricted to a younger island (or islands) [62]. For this reason, applying island calibrations to species that predominantly have widespread ranges can be difficult because such patterns are not always apparent. However, the Hawaiian *Dicranomyia* do have two groups that show clear and well-supported splits between old and young island taxa (*D. jacobus/grimshawi* and *D. variabilis/kraussi/stygipennis*: Figure 1).

**Figure 2:**
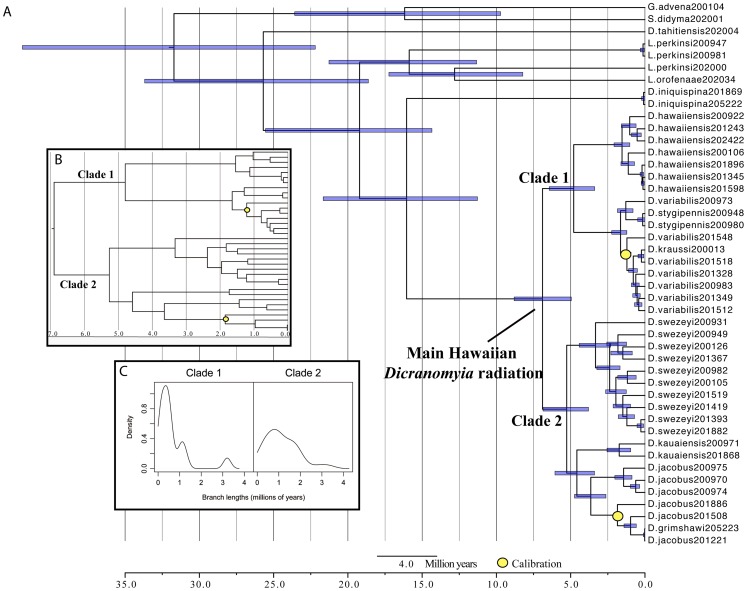
Time tree with error bars, Island Calibrations II. A. main figure: purple bars are 95%HPD of node age estimates. *D. variabilis* calibration  = (1.3 my SD = 0.15), *D. jacobus* calibration  = (1.9 my, SD = 0.15); B. Inset: figure is a closeup of the ingroup, showing the disparity in branch lengths among Clade 1 and Clade 2; C. Inset: a comparison of branch length distributions between Clade 1 and Clade 2.

One node from each of these groups was calibrated (shown on Figure 2), using normally distributed priors with the means set on the age thought to be most relevant for biota – the end of shield building [63], and standard deviations set to accommodate uncertainty as to when the taxa colonized the islands. The island calibration for each node was selected using the ancestral range reconstruction method described in the following section (unconstrained model). In the case of *D. variabilis*, the ancestral range was reconstructed most strongly as Hawaii, but ranges of Maui or Maui and Hawaii were also assigned significant weight (Table 2). In the case of *D. jacobus*, the reconstruction was Molokai or Molokai and Hawaii (Table 2). In order to accommodate uncertainty in the reconstruction of the *D. variabilis* node, two divergence time analyses were run: Island Calibrations I used Hawaii's age for *D. variabilis* (0.5 my, SD = 0.15), Island Calibrations II used Maui's age (1.3 my, SD = 0.15). Both analyses used the age of Molokai for the *D. jacobus* node (1.9 my, SD = 0.15).

**Table 2:** Divergence times and biogeography.

Node	Divergence time estimates: (95% HPD)				Ancestral range reconstruction Unconstrained Model (U)
	Calibration based estimates		Rates based estimates		
	Island Calibrations I	Island Calibrations II	Rates I 5.2%/my	Rates II 2.3%/my	
*D. iniquispina/main Hawaiian radiation*	12.58	16.05	11.63	16.63	
	(8.47–17.07)	(11.28–21.66)	(6.32–22.27)	(8.05–29.98)	
**MRCA of main Hawaiian** ** **radiation**	**5.37**	**6.90**	**5.25**	**7.73**	0.45 widespread, 0.11 Kauai, 0.09 Oahu, 0.10 Molokai,
	**(3.87–7.28)**	**(4.96–8.8)**	**(2.93–9.28)**	**(3.93–13.40)**	0.08 Maui, 0.13 Hawaii
**Clade 1**					
*D. variabilis/D. hawaiiensis*	3.76	4.80	3.83	5.6	0.33 Hawaii, 0.24 widespread, 0.21 Kauai, 0.07 Oahu,
	(2.45–5.30)	(3.39–6.44)	(2.05–7.05)	(2.79–10.04)	0.06 Molokai, 0.04 Maui
*D. variabilis* (with* D. stygipennis* and *D. Kraussi*)	1.21	1.64	1.26	1.78	0.32 Hawaii, 0.30 widespread, 0.20 Kauai, 0.08 Oahu,
	(0.79–1.77)	(1.22–2.24)	(0.57–2.28)	(0.88–3.12)	0.02 Molokai, 0.04 Maui
*D. variabilis* (with* D. stygipennis*)	0.95	1.29	1.00	1.35	0.65 widespread, 0.16 Kauai, 0.14 Oahu, 0.007 Hawaii
	(0.59–1.41)	(0.80–1.85)	(0.43–1.91)	(0.62–2.61)	
*D. variabilis* (with *D. kraussi*)*	0.53	1.21	0.74	1.10	0.66 Hawaii, 0.17 Maui/Hawaii, 0.12 Maui
	(0.44–0.85)	(0.94–1.49)	(0.34–1.42)	(0.45–2.02)	
*D. hawaiiensis*	1.23	1.54	1.21	1.75	0.40 Hawaii, 0.23 widespread, 0.20 Kauai, 0.02 Oahu,
	(1.75–1.72)	(1.02–2.08)	(0.61–2.44)	(0.87–3.35)	0.08 Molokai, 0.03 Maui
**Clade 2**					
*D. swezeyi/D. kauaiensis/ D. jacobus*	4.19	5.27	4.25	6.21	0.62 widespread, 0.10 Kauai, 0.09 Oahu, 0.06 Molokai,
	(2.97–5.55)	(3.80–6.86)	(2.19–7.35)	(3.23–10.82)	0.02 Maui, 0.06 Hawaii
*D. swezeyi*	2.67	3.33	2.65	3.88	0.49 widespread, 0.16 Kauai, 0.08 Oahu, 0.03 Molokai,
	(1.71–3.65)	(2.31–4.42)	(1.44–4.68)	(1.89–6.68)	0.10 Maui, 0.09 Hawaii
*D. kauaiensis/D. jacobus*	3.69	4.58	3.56	5.20	0.32 Molokai, 0.26 widespread, 0.21 Oahu, 0.10 Kauai,
	(2.65–4.89)	(3.40–6.06)	(2.00–6.33)	(2.70–8.91)	0.004 Maui, 0.06 Hawaii

Node age estimates from Beast analyses are shown; estimates based on island calibrations are presented in columns 2 and 3, estimates based on a divergence rates are presented in columns 4 and 5. Ancestral range reconstructions from are shown on the far right in column 6. We have simplified the Lagrange results by summarizing the probabilities for reconstructions that occurred across multiple islands, naming them “widespread”. Results with the highest probabilities are underlined. Asterisk indicates that the node was used as a calibration in the calibration based analyses.

While island calibrations have been widely used in Hawaiian lineages (e.g., [1], [64]) there are several caveats to their application that should be considered. For example, it is plausible that divergence among populations occurred prior to island emergence and was thus unrelated [65]. Furthermore, among species with such widespread ranges as *Dicranomyia*, it is conceivable that present and past movement among islands has obscured the true biogeographic history. In that case, the distribution patterns and ancestral state reconstructions would be skewed. For this reason, we sought to use an alternative method to estimate divergence dates in order to provide a comparison.

#### Divergence rate

An alternative approach to divergence date estimation is to use locus-specific rates. We performed two analyses using COI rates from the literature, applying the rates as a prior to the COI partition and allowing rates of the other partitions to vary. The mitochondrial gene COI has been used extensively to estimate divergence times among arthropod taxa (e.g.: [66], [67], [68]) and researchers are accustomed to thinking about molecular rates based on this gene. However, the difficulty in applying divergence rates is in selecting which rates to use, since this parameter is unknown for most species.

In the first analysis (Rates I), a COI divergence rate derived from Hawaiian arthropods, 5.2%/million years, was applied as a normal prior to the ucld.mean parameter of the COI partition (ucld.mean, x = 0.026, SD = 0.007). This rate was based on divergence among pairs of taxa that are situated with one taxon on Maui and the other on Hawaii, and includes a wide variety of arthropods including moths, beetles, flies and spiders (data is from Table S3 within reference [69]). The uncorrected pairwise sequence divergence was calculated for each of the pairs of taxa, outliers were excluded, and an average was taken. This average was divided by the age when Hawaii reached its maximum height, approximately 0.5 million years ago [63], which tends to be considered the most biologically plausible scenario for most taxa that rely on having mature habitats in place before being able to establish. In the second analysis (Rates II), a commonly applied divergence rate for the COI locus in arthropods (2.3%/million years [68]) was applied as a normal prior to the ucld.mean parameter of the COI partition (ucld.mean, x = 0.0115, SD = 0.0068).

The same 5 gene concatenated data set (COI, COII, ND2, 16s and CAD) including all individuals was analyzed in each of the four analyses (Island Calibrations I and II; Rates I and II). Partitions and the best fit models of evolution for each partition were selected using BIC in PartitionFinder [55]. Partitioning in the divergence rate analysis differed only slightly from the island calibration analysis in that COI was assigned its own partition (Appendix S3 in File S1). Site and clock models were unlinked and all partitions were analyzed using the uncorrelated lognormal relaxed clock except for the partitions including 16S, for which a strict clock could not be rejected and was thus applied. The tree-shape prior was linked across partitions and specified as a Yule Process. Base frequencies were estimated from the data and a starting tree was generated in RAxML [60]. Six independent MCMC searches were conducted, each running for 100 million generations and sampled every 1000 generations. The number of generations were selected to generate Effective Sample Sizes (ESS) values greater than 200 for each of the parameters [70]. Convergence was assessed using Tracer v. 1.7.2 and trees were summarized using Tree Annotator v. 1.7.2 after removing trees from the burn-in phase.

Pairwise distances were calculated on the time-calibrated tree using the cophenetic.phylo function in APE [71], performed in R [72]. Differences in branch length distributions among Clades 1 and 2 were assessed by testing for differences in the skewness of each clade. We performed 1000 permutations of the branch length distributions of each clade to create a null distribution of differences in skewness and tested the observed difference in skewness to the 95% quantile of this permutational null distribution.

### Ancestral Range Reconstruction

The historical biogeographic ranges of the main Hawaiian *Dicranomyia* radiation were estimated using the maximum likelihood Dispersal-Extinction-Cladogenesis ancestral range reconstruction method in Lagrange v.2 [73]. This method was selected because it allows for the evaluation of changing geology over time and generates estimates of uncertainty in ancestral ranges. Furthermore, it models peripheral isolate speciation [73], which has been suggested to be important to the evolution of Hawaiian taxa [74]. Reconstructions were conditioned in absolute time with the dated phylogeny from Beast (Island Calibrations II). A five-state model was used, including Kauai, Oahu, Molokai, Maui and Hawaii Island. *Dicranomyia iniquispina*, was used as the outgroup. Two ancestral range models were tested: 1) unconstrained and 2) time-stratified. Because there is uncertainty between the divergence time analyses, the unconstrained model was used in order to assess the strength of the biogeographic signal in the data without any constraints. The time-stratified model did not allow lineages to colonize islands before their emergence [63]. In all models, transitions rates between coded regions were set as equal. Model performance was assessed by selecting the reconstruction method that yielded the highest log-likelihood value (ensuring that at least 2 log-likelihood units separated the top model from the others), while considering the results in the geologic context [75].

## Results

### Phylogenetic Relationships

Tree topologies generated by analyses of different individual loci or by different optimality criteria for each locus were very similar, although levels of support and resolution varied based on the size of the data set and numbers of informative characters (Appendices S4a-f in File S1). Likewise, tree topologies generated using ML and BI approaches of the concatenated data set were very similar and, at well-supported nodes, they were identical. Figure 1 is the BI consensus tree showing both ML and BI support values (see Appendix S5 in File S1 for the ML topology). Basal nodes are generally well supported (Figure 1). However, the BI and ML analyses disagree with respect to the placement of *Libnotes* and *D. iniquispina*. While the BI analysis resulted in a polytomy containing *Libnotes*, *D. iniquispina* and the rest of the Hawaiian *Dicranomyia* (Figure 1), the ML analysis showed some support for a sister relationship of *D. iniquispina* with the rest of the Hawaiian *Dicranomyia* (BS = 73: Appendix S5 in File S1). There is strong support for a radiation of *Dicranomyia* within Hawaii (BS = 100, PP = 100) exclusive of *D. iniquispina* and we will focus on this lineage in the present paper.

The radiation within Hawaii is split into two major clades: (1) *D. hawaiiensis* + *D.variabilis* + *D. krausii* + *D. stygipennis* (BS = 100, PP = 100) and (2) *D. sweyzeyi + D. kauaiensis* + *D. jacobus* + *D. grimshawii* (BS = 100, PP = 100: Figure 1). Within these clades, there is good support for the monophyly of *D. hawaiiensis, D. stygipennis, D. swezeyi* and *D. kauaiensis. D. variabilis* is not monophyletic; however, together *D. variabilis, D. kraussi* and *D. stygipennis* form a well-supported monophyletic group. *D. grimshawi* is nested within *D. jacobus*, but there is strong support for a monophyletic grouping of the two (Figure 1).

### Divergence Time Estimation

The topologies generated from the divergence time analyses are identical to the topology in Figure 1 with one exception: all four analyses show strong support for a sister relationship between *D. iniquispina* and the rest of the Hawaiian *Dicranomyia* (PP = 98–100). The chronograms are shown in Figure 2 and Appendices S6a-c in File S1.

The Island Calibrations I and Rates I analyses resulted in similar node age estimates; the median age for the MRCA of the main Hawaiian radiation was estimated in both analyses at approximately 5 million years, while the estimate for the age of the MRCA of *D. iniquispina* and the main Hawaiian radiation of *Dicranomyia* was approximately 12 million years (Table 2). The Island Calibrations II and Rates II analyses also yielded very similar estimates; the median age for the MRCA of the main Hawaiian radiation was estimated as approximately 7 million years, while the estimate for the age of the MRCA of *D. iniquispina* and the main Hawaiian radiation of *Dicranomyia* was approximately 12 million years (Table 2). In all cases, the 95% HPDs are wider for the rates-based analyses than the calibration-based analyses. Estimates for all main nodes are presented in Table 2, Figure 2 and Appendices S6a-c.

The difference between Islands Calibrations I and Islands Calibrations II is that the former used Hawaii's age as the calibration for *D. variabilis + D. kraussi*, while the latter used Maui's age. There is support for either Hawaii (prob = 0.66) or Maui and Maui + Hawaii (prob = 0.29) as the ancestral range for the calibrated node (Table 2). While Hawaii has a higher probability of being the ancestral range given this reconstruction, this could simply be an effect of sampling, and because of this, we ran the analysis both ways. Where both calibrations are possibilities, assuming a MRCA on Maui is more conservative because it results in the upper bounds of possible age estimates. Likewise, the COI rate of 2.3% per million years used in the Rates II analysis is the more conservative rates-based estimate, and Island Calibrations II and Rates II corroborate one another well. Because of this, the results from Island Calibrations II are presented in Figure 2 and discussed below. The median colonization time for the main radiation of *Dicranomyia* is estimated at 6.9 million years ago (4.96–8.8 95% HPD, Island Calibrations II, Table 2), prior to the emergence of Kauai. However, the 95% HPD interval very slightly overlaps the age of Kauai, so the possibility that the ancestor arrived to Kauai cannot be excluded. Together, Island Calibrations I and Rates I also corroborate one another well and provide the younger bounds for *Dicranomyia* in the Hawaiian Islands (Table 2, Appendices S6a and S6b).

Although the crown groups within Clade 1 and Clade 2 both originate in the same time period (4.5–5.5 million years ago), species within Clade 1 are much younger. Pairwise distances in units of million years in the two main clades and within species are as follows: Clade 1: 0.31–9.59; *D. variabilis* (+ *D. krausii* + *D. sygipennis*): 0.31–3.29; *D. hawaiiensis*: 0.39–3.08; Clade 2: 0.04–10.53; *D. kauaiensis:* 3.46; *D. swezeyi*: 0.55–6.65; and *D. jacobus* (+*D. grimshawi*): 0.04–7.29. The distribution of branch lengths within Clade 1 is significantly more skewed toward the present relative to Clade 2 (observed value = 1.59; null distribution 95% confidence interval  = [–1.177, 1.11]; Figure 2c).

### Ancestral Range Reconstruction

**Figure 3:**
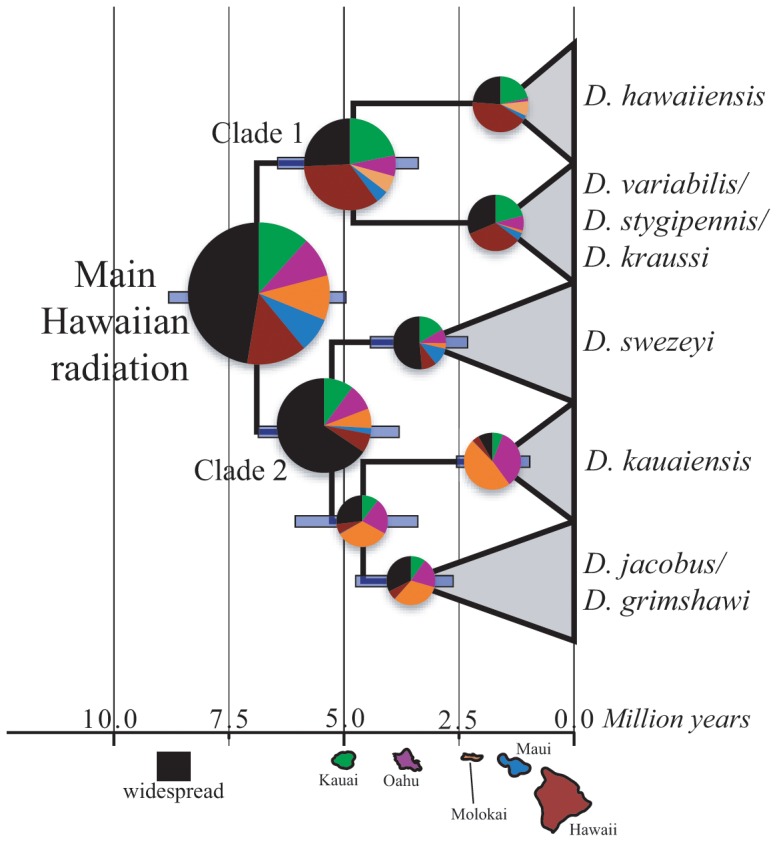
Results of divergence dating analysis and ancestral range reconstruction. The Maximum Clade Credibility tree is shown compared to a timescale bar, with populations collapsed into species. Outgroup has been trimmed.

Overall likelihood scores (–lnL) for each model were as follows: U = –94.41 and TS- = –119.7. The unconstrained model clearly outperformed the time stratified model, and its reconstructions are presented in Table 2 and Figure 3. However, the unconstrained model results must be interpreted carefully because of the timing of the appearance of the islands. Although the timing of the islands was explicitly considered in the time stratified model, the very large difference in likelihood scores between this model and the unconstrained model indicates a poor fit to the data. Thus, we present the unconstrained model results here.

As discussed in the introduction, the *Dicranomyia* are characterized by a low rate of single island endemism relative to the Hawaiian *Drosophila* and some other endemic Hawaiian radiations (30% vs. >90%, [25], [26]). The presence of single species on multiple islands yields ancestral reconstructions that span multiple islands. For simplicity, we summarize all reconstructions that are spread over multiple islands as widespread, except in cases where one particular multi-island range has a large probability assigned to it.

The ancestral reconstruction for the root of the *Dicranomyia* radiation on Hawaii was reconstructed as widespread (prob = 0.45), consisting of 26 different multi-island range configurations, with the remaining probability fairly evenly split among all of the islands (0.08–0.13). The ancestral range for the most recent common ancestor (MRCA) of Clade 1 was equivocal between Kauai, Hawaii and widespread (prob = 0.21, 0.33 and 0.24), as was *D. hawaiiensis* (prob = 0.21, 0.40, 0.23) and *D. variabilis* (+*D. krausi + D. stygipennis*) (prob = 0.20, 0.32, 0.30). The ancestral range for *D. variabilis + D. kraussi* was reconstructed as Hawaii or Maui and Hawaii (prob = 0.66, 0.29). The ancestral range for Clade 2 was reconstructed as widespread (prob = 0.62), as was the range for *D. swezeyi* (prob = 0.49). The MRCA of *D. kauaiensis* and of *D. jacobus* was reconstructed as Molokai and widespread (prob = 0.32, 0.26, respectively). The MRCA of *D. kauaiensis* was reconstructed as Molokai or Oahu (prob = 0.45, 0.32) and of *D. jacobus* was reconstructed as Molokai and widespread (prob = 0.30, 0.31, respectively). Finally, the MRCA for the young island *D. jacobus* was reconstructed as Molokai or Molokai and Hawaii (prob = 0.60) (Table 2, Figure 3). All of these same nodes had ancestral ranges reconstructed as Kauai under the time-stratified model.

## Discussion

### Taxonomic implications

Results from this study indicate the need for further taxonomic study of the Hawaiian *Dicranomyia*. While most nodes in the tree are very well supported and several of the original species appear to be good species and are monophyletic, there are two species clusters that will require additional taxonomic study. In each case, voucher specimens were re-examined and were found to have been accurately identified according to their morphological descriptions. First, our analyses place *D. grimshawi* within *D. jacobus*, (BS = 100, P = 100: Fig. 1), rendering the latter taxon paraphyletic, in spite of the fact that these taxa are morphologically distinct. Another species, *D. variabilis,* is paraphyletic with respect to *D. kraussi* (BS = 97, PP = 100: Fig. 1) and *D. stygipennis* (BS = 69, PP = 100: Fig. 1). These three species are closely related morphologically and the discordance in the molecular data may be the result of an incomplete lineage sorting event or current or past gene flow. Future morphological and genetic work should focus on examining multiple individuals of *D. grimshawi* to better understand its relationship to *D. jacobus* and on clarifying the relationship between *D. variabilis, D. kraussi* and *D. stygipennis*.

### Biogeography

Nitta and O'Grady (2008) suggested that *D. iniquispina* may be the result of a separate colonization to the Hawaiian Islands from the rest of the Hawaiian *Dicranomyia*. While its position relative to the rest of the Hawaiian *Dicranomyia* is equivocal in our BI analysis (Figure 1), both the ML analysis and the Bayesian divergence time analyses recover topologies that show support for a sister relationship for *D. iniquispina* with the main Hawaiian radiation, suggesting a single colonization event. However, it is distinct, both in terms of its genital morphology and genetics. Hardy [29] considered *D. iniquispina* a relative of *D. grimshawi*, differing mainly in the number of strong spines on the ventral prolongation of the ventral dististylus. He also stated that the “differences in the shapes of the ventromesal lobes of the basistyli and the posterior margin of the ninth tergum. are also significant.” It is clear from our analyses that *D. iniquispina* is quite distantly related to *D. grimshawi* and the other Hawaiiana craneflies. Furthermore, *D. iniquispina* resides on a very long branch and was equally divergent in the BI analyses (Fig. 1) from the other Hawaiian endemic taxa as was the genus *Libnotes*. The median age estimate for the MRCA of *D. iniquispina* and the main Hawaiian radiation range between approximately 11.5 and 16.5 million years in the four divergence time analyses (Table 2), a significant increase over the age of the other Hawaiian taxa in clades 1 and 2 (min = 5.37, max = 7.73 million years). It is possible that they are derived from the same colonization event – this would have involved colonization to an older, now eroded island followed by colonization to the current high Hawaiian Islands 5–8 million years later (Table 2), possibly with several extinction events in intervening taxa to generate the long branches we observe in the extant species. Alternatively, it is plausible that the archipelago was colonized two times by the genus *Dicranomyia,* once by *D. iniquispina* and again by the ancestor of the other *Dicranoymia* species. The current data do not allow us to differentiate between these two hypotheses and further research, sampling this genus across the Pacific, is needed to resolve this question.

The median colonization time for the main radiation of *Dicranomyia* is estimated at 6.9 million years ago (4.96–8.8 95% HPD, Table 2: Island Calibrations II). This is prior to the emergence of Kauai, about the time when Nihoa, one of the now-eroded Northwest Hawaiian Islands, was at its maximum height of 1300 m [76], 7.2 million years ago [63]. Unfortunately, it is not possible to include contemporary taxa from Nihoa or any of the other formerly high islands as these highly eroded islands no longer contain suitable habitat for *Dicranomyia* and no species from this genus have been recorded from islands older than Kauai [25].

The range reconstruction for the MRCA of the main Hawaiian *Dicranomyia* radiation is widespread (Table 2, Figure 3), which is not surprising given that many of the modern Hawaiian *Dicranomyia* are found on one or more islands. However, the reconstruction for this node is widespread across the contemporary high islands, which would not be possible given the divergence time estimate and emergence dates of the high Hawaiian islands. In the Lagrange analysis, this issue was handled by comparing the unconstrained model to a time-stratified model that did not allow the taxa to colonize each island prior to its formation. The two models returned results at almost opposite ends of the possible spectrum. The time stratified model reconstructed most of the ancestral ranges at the species level and above as Kauai. The unconstrained model, on the other hand, reconstructed many of these same nodes as occurring on multiple islands. There is more certainty in the reconstructions towards the tips of the tree in the unconstrained model (*e.g.*, within *D. jacobus* and *D. variabilis*), but the biogeographic signal decays rapidly as you move back in time, most likely due to past and present movement among islands.

What do the ancestral range reconstructions and divergence time estimates tell us about the biogeography of *Dicranomyia* in the context of the dynamic Hawaiian Islands? The divergence time estimates indicate that diversification has been occurring in the *Dicranomyia* more or less continuously since the colonization of the islands by the MRCA of the main radiation (Figure 2). It is difficult to imagine that all of this diversification occurred on Kauai, as is indicated by the time-stratified model, given the wide distributions of the contemporary taxa. However, the better-performing unconstrained model has generated range estimates on islands that did not exist at the time of the diversification event, which seems equally unlikely. We propose, based on the divergence time estimates and biogeographic analyses, that once new species of *Dicranomyia* formed they rapidly moved between whatever islands existed at the time. This effectively obscured any biogeographic signal. Therefore, at least in *Dicranomyia*, ancestral range reconstructions deeper than the tips of the tree are likely to show little of the biogeographic history of this group.

Although the crown groups within Clade 1 and Clade 2 originate in roughly the same timeframe 4.5–5.5 million years ago, a clear difference is apparent between them in their distributions of branch lengths (inset, Figure 2b,c) and pairwise phylogenetic distances. Species in Clade1 are much younger, and the topology within this clade is characterized by long branches leading to a near-simultaneous burst of diversification around 1.5 million years ago in its two separate lineages, in the timeframe of Maui Nui [63] (Figure 2b). In contrast, the diversity within Clade 2 is much older and it appears that diversification has been occurring more evenly over time since it originated (Figure 2b). Why the two are so different is not clear, and may be a result of differing speciation or extinction rates among the two clades.

Apart from the MRCA of the main radiation in the islands, the remaining node estimates fall comfortably within the timeframe of the contemporary high islands (Table 2). The natural history of the group in Hawaii supports these timing results demonstrating that substantial diversification has occurred within the context of the current high islands. There are three species of endemic flightless *Dicranomyia* known from the high elevation terrain of Kauai and Oahu (*D. sabroskyana* Byers 1982 [34] from Kauai, *D. hardyana Byers* 1985 [33] from Oahu and *D. gloria* Byers 1994 [32] from Oahu). Although they were not included in this study, they are still interesting because a transition to flightlessness would, by definition, restrict dispersal and is typically thought of as an island syndrome that evolves *in situ* [77].

Several present-day *Dicranomyia* have range distributions span multiple islands, indicating that dispersal between neighboring islands is fairly easy. But if they are able to disperse so well, what would cause speciation in this group? A history in the archipelago that extends back prior to the emergence of Kauai would have involved numerous inter-island colonization events as the ancestral taxa navigated the repeated appearance and disappearance of islands over time, and would also have involved stochastic extinction as some lineages died out with disappearing habitat. This would have pruned out some of the diversity in the group, and potentially is what gave rise to the long branches observed in Clade 1. Certainly divergence in allopatry, most likely induced when ranges became disjunct via dispersal and extinction, played a role in divergence of this group. However, because it is known that larval *Dicranomyia* make use of a diverse range of habitats, future work that focuses on the ecological requirements of the immature stages of each species would be an interesting complement to the biogeographic and temporal patterns established here and may provide insight into the role of ecological diversification in this group.

## Supporting Information

File S1 **Appendix S1, Collection Information and Genbank Accessions of Species Included in the Present Study. Appendix S2, Primer names and references. Appendix S3, Summary of data partitions and the nucleotide models determined for each partition. Appendix S4, Individual Bayesian Gene Trees. Appendix S5, Combined maximum likelihood analysis of all genes. Appendix S6, Alternate BEAST Trees.**
(DOCX)
