# Supplementary material for: Molecular Phylogeny and Biogeography of the Hawaiian Craneflies Dicranomyia (Diptera: Limoniidae)
Source: PLoS One. 2013 Sep 13;8(9):e73019. doi: 10.1371/journal.pone.0073019 (PMC3772799; doi:10.1371/journal.pone.0073019)
Supplement: File S1 — Appendix S1, Collection Information and Genbank Accessions of Species Included in the Present Study. Appendix S2, Primer names and references. Appendix S3, Summary of data partitions and the nucleotide models determined for each partition. Appendix S4, Individual Bayesian Gene Trees. Appendix S5, Combined maximum likelihood analysis of all genes. Appendix S6, Alternate BEAST Trees. (DOCX) [file pone.0073019.s001.docx]

**Supplementary information for:** Molecular phylogeny and biogeography of the Hawaiian craneflies *Dicranomyia* (Diptera: Limoniidae)

Kari Roesch Goodman and Patrick O’Grady

**Supplementary Inventory:**

Appendix S1. Collection Information and Genbank Accessions of Species Included

Appendix S2. Primer names and references

Appendix S3. Summary of data partitions and nucleotide models

Appendix S4a-f. Individual Bayesian Gene Trees

Appendix S5. Combined maximum likelihood analysis of all genes

Appendix S6a-c. Alternate BEAST Trees.

**Appendix S1**. Collection Information and Genbank Accessions of Species Included in the Present Study.

**Ingroup**

*Dicranomyia grimshawi (Alexander, 1919)*

HAWAIIAN ISLANDS: HAWAI’I; Hawaii Volcanoes National Park, Ola’a Forest, pole 44; 28vii2010; O’Grady, Lapoint, Ort; 205223 [16S: KC692801; CO1: KC692791; COII: KC692796; ND2: KC692802; CAD: KC692815]

*Dicranomyia hawaiiensis (Grimshaw, 1901)*

HAWAIIAN ISLANDS: KAUA’I: Pu’u Ka Pele Forest Reserve, Papa’ala’i Ditch Trail, sweeping under Pisonia; 1.iii.2006; O’Grady & Bennett; 200922 [16S: EU005404; COI: EU005439; ND2: EU005517; CAD: KC692826]

HAWAIIAN ISLANDS: MOLOKA’I: Kamakou Forest Preserve, Makai of Kolekole Cabin on 4WD Road; 19.ii.2007; O’Grady, Magnacca, Lapoint & Bennett; 201896 [16S: EU005406; COI: EU005443; COII: EU005481; ND2: EU005519]

HAWAIIAN ISLANDS: MOLOKA’I: Kamakou Forest Preserve, Makai of Kolekole Cabin on 4WD Road; 19.ii.2007; O’Grady, Magnacca, Lapoint & Bennett; 202422 [COI: EU005444; COII: EU005482; CAD: KC692825]

HAWAIIAN ISLANDS: MAUI: Waikamoi Forest Preserve, stream from flume; 4.viii.2005; O’Grady, Bennett, Gatesy & Hayashi; 201345 [COI: EU005441; COII: EU005479; ND2: EU005518]

HAWAIIAN ISLANDS: HAWAI’I: Kohala Mountains, Top of Waipio Falls; O’Grady, Specht, Hotchkiss, Schuurman & Giannullo; 201598 [16S: EU005405; COI: EU005442; COII: EU005480; CAD: KC692827]

HAWAIIAN ISLANDS: HAWAI’I: Hawaii Volcanoes National Park, Top of Mauna Loa Road; 11.xii.2003; O’Grady, 200106 [16S: EU005403; COI: EU005438; COII: EU005477; ND2: EU005516; CAD: KC692828]

HAWAIIAN ISLANDS: HAWAI’I: Saddle Road, Powerline Trail, Deep kipuka; 12.viii.2005; O’Grady, Magnacca & Bennett; 201243 [COI: EU005440; COII: EU005478]

*Dicranomyia iniquispina (Hardy, 1953)*

HAWAIIAN ISLANDS: MOLOKA’I: Pu’u Kolekole, 3854 ft; 19.ii.2007; O’Grady, Magnacca, Lapoint, Bennett; 201869 [16S: EU005407; COI: EU005445; COII: EU005483]

HAWAIIAN ISLANDS: HAWAI’I; Hawaii Volcanoes National Park, Ola’a Forest, pole 44; 28vii2010; O’Grady, Lapoint, Ort; 205222 [16S: KC692800; CO1: KC692793; CO2: KC692797; CAD: KC692800]

*Dicranomyia jacobus (Alexander, 1919)*

HAWAIIAN ISLANDS: KAUA’I: Na Pali Kona Forest Preserve, Pihea Trail; 28.ii.2006; O’Grady & Bennett; 200975 [16S: EU005409; COI: EU005448; COII: EU005486; ND2: EU005522; CAD: KC692813; SNF: KC924731]

HAWAIIAN ISLANDS: KAUA’I: Pu’u Ka Pele Forest Reserve, Papa’ala’i Ditch Trail, sweeping under Pisonia; 1.iii.2006; O’Grady & Bennett; 200974 [16S: EU005408; COI: EU005447; COII: EU005485; ND2: EU005521; CAD: KC692811; SNF: KC924730]

HAWAIIAN ISLANDS: O’AHU: Waianae-Kaala Trail, 2300 ft.; 5.iii.2006; O’Grady, Magnacca & Bennett; 200970 [COI: EU005446; COII: EU005484; ND2: EU005520; CAD: KC692812; SNF: KC924729]

HAWAIIAN ISLANDS: MOLOKA’I: Kamakou Forest Preserve, Pu’u Kolekole, 3854 ft; 19.ii.2007; O’Grady, Magnacca, Lapoint, Bennett; 201886 [16S: EU005411; COI: EU005449; COII: EU005488; CAD: KC692816; SNF: KC924733]

HAWAIIAN ISLANDS: HAWAI’I; Hawaii Volcanoes National Park, Ola’a Forest, right side of road at turnaround; 11.viii.2005; O’Grady & Bennett; 201221 [COI: KF214459; COII: EU005487]

HAWAIIAN ISLANDS: HAWAI‘I: Pu’u Makaala Forest Reserve, near Stainback Highway,; 11.vii.2004; O’Grady & Giannullo; 201508 [16S: EU005410; COI: KC692794; COII: KC692798; CAD: KC692814; SNF: KC924732]

*Dicranomyia kauaiensis (Grimshawi, 1901)*

HAWAIIAN ISLANDS: O’AHU: Waianae-Kaala Trail, 2300 ft.; 5.iii.2006; O’Grady, Magnacca & Bennett; 200971 [16S: EU005412; COI: EU005450; COII: EU005489; ND2: EU005523; CAD: KC692810; SNF: KC924734]

HAWAIIAN ISLANDS: MOLOKA’I: Kamakou Forest Preserve, near Hanalilolilo Lookout; 19.ii.2007; O’Grady, Magnacca, Lapoint & Bennett; 201868 [16S: EU005414; COI: EU005451; COII: EU005490; ND2: EU005524; CAD: KC692809; SNF: KC924735]

*Dicranomyia kraussi (Alexander, 1951)*

HAWAIIAN ISLANDS: MAUI: Waikamoi Forest Preserve, Pig Hunter’s Trail; 16.xii.2003; O’Grady; 200013 [16S: EU005415; COI: EU005452; COII: EU005491; ND2: EU0055205; CAD: 200013; SNF: KC924737]

*Dicranomyia stygipennis (Alexander, 1919)*

HAWAIIAN ISLANDS: O’AHU: Waianae-Kaala Trail, 2300 ft.; 5.iii.2006; O’Grady, Magnacca & Bennett; 200948 [16S: EU005417; COI: EU005453; COII: EU005492; CAD: KC692823]

HAWAIIAN ISLANDS: O’AHU: Kuliouou Gulch; 10.vi.2005; Magnacca; 200980 [16S: EU005418; CAD: KC692822]

*Dicranomyia swezeyi (Alexander, 1919)*

HAWAIIAN ISLANDS: KAUA’I: Powerline Trail, end of Kapa Ka Road; 2.ii.2006; O’Grady & Bennett; 200931 [16S: EU005421; COI: EU005456; COII: EU005495; CAD: KC692807; SNF: KC924739]

HAWAIIAN ISLANDS: O’AHU: Waianae-Kaala Trail, 2300 ft.; 5.iii.2006; O’Grady, Magnacca & Bennett; 200949 [16S: EU005422; COI: EU005457; COII: EU005496; ND2: EU005528; CAD: KC692806; SNF: KC924740]

HAWAIIAN ISLANDS: MOLOKA’I: Kamakou Forest Preserve, Pu’u Kolekole, 3854 ft; 19.ii.2007; O’Grady, Magnacca, Lapoint, Bennett; 201882 [16S: EU005424; COI: EU005462; COII: EU005497; CAD: KC692803; SNF: KC924742]

HAWAIIAN ISLANDS: MAUI: Waikamoi Forest Preserve, Heed Trail; 5.viii.2005; O’Grady, Bennett, Gatesy & Hayashi; 201419 [COI: EU005461; COII: EU005500]

HAWAIIAN ISLANDS: MAUI: Waikamoi Forest Preserve, Pig Hunter’s Trail; 16.xii.2003; O’Grady; 200126 [16S: EU005420; COI: EU005455; COII: EU005494; ND2: EU005527; CAD: KC692804; SNF: KC924738]

HAWAIIAN ISLANDS: MAUI: Waikamoi Forest Preserve, Pig Hunter’s Trail; 5.viii.2005; O’Grady, Bennett, Gatesy & Hayashi; 201393 [COI: EU005460; COII: EU005499]

HAWAIIAN ISLANDS: MAUI: Waikamoi Forest Preserve, Heed Trail; 4.viii.2005; O’Grady, Bennett, Gatesy & Hayashi; 201367 [COI: EU005459; COII: EU005498; ND2: EU005530]

HAWAIIAN ISLANDS: HAWAI’I: Hamakua Coast, Kalopa State Park; 13.xii.2003; O’Grady & Foote; 200105 [16S: EU005419; COI: EU005454; COII: EU005493; ND2: EU005526]

HAWAIIAN ISLANDS: HAWAI’I: Hawaii Volcanoes National Park, Kipuka Puaulu; 19.vi.2006; Magnacca; 200982 [16S: EU005423; COI: EU005458; COII: EU005501; ND2: EU005529; CAD: KC692805; SNF: KC924741]

HAWAIIAN ISLANDS: HAWAI’I: Hilo Forest Reserve, Saddle Road, ~mile 16; 12.vii.2004; O’Grady & Giannullo; 201519 [16S: EU005413; COI: KC692795; COII: KC692799; CAD: KC692808; SNF: KC924736]

*Dicranomyia variabilis (Grimshaw, 1901)*

HAWAIIAN ISLANDS: KAUA’I: Pu’u Ka Pele Forest Reserve, Papa’ala’i Ditch Trail, sweeping under Pisonia; 1.iii.2006; O’Grady & Bennett; 200973 [16S: EU005426; COI: KF214460; COII: EU005503; ND2: EU005532; CAD: KC692824]

HAWAIIAN ISLANDS: MAUI: Waikamoi Forest Preserve, Pig Hunter’s Trail; 5.viii.2005; O’Grady, Bennett, Gatesy & Hayashi; 201328 [COI: EU005465; COII: EU005505; ND2: EU005533]

HAWAIIAN ISLANDS: MAUI: Waikamoi Forest Preserve, stream from flume; 4.viii.2005; O’Grady, Bennett, Gatesy & Hayashi; 201349 [COI: EU005466; COII: EU005506; ND2: EU005534]

HAWAIIAN ISLANDS: HAWAI’I: Hilo Forest Reserve, Saddle Road, ~mile 16; 12.vii.2004; O’Grady & Giannullo; 201518 [16S: EU005429; COI: EU005468; COII: EU005507; CAD: KC692819; SNF: KC924745]

HAWAIIAN ISLANDS: HAWAI’I: Hilo Forest Reserve, Saddle Road, ~mile 12; 12.vii.2004; O’Grady & Giannullo; 201512 [16S: EU005428; COI: EU005467; CAD: KC692821; SNF: KC924744]

HAWAIIAN ISLANDS: HAWAI’I: Hawaii Volcanoes National Park, Kipuka Puaulu; 19.vi.2006; Magnacca; 200983 [16S: EU005427; COI: EU005464; COII: EU005504]

HAWAIIAN ISLANDS: HAWAI’I: Saddle Road, Pu’u O’o Trail, Kipuka 2, 5880 ft.; 15.vii.2005; O’Grady & Giannullo; 201548 [16S: EU005430; CAD: KC692817]

**Outgroups**

*Dicranomyia tahitiensis (Alexander, 1921)*

FRENCH POLYNESIA: MOOREA: Trail to Afaraitu Cascade; 11.vii.2006; O’Grady, Evenhuis & Hembry; 202004 [16S: EU005425; COI: EU005463; COII: EU005502; ND2: EU005531; CAD: KC692831; SNF: KC924743]

*Libnotes perkinsi (Grimshaw, 1901)*

HAWAIIAN ISLANDS: HAWAI’I: Hawaii Volcanoes National Park, Kipuka Puaulu; 19.vi.2006; Magnacca; 200981 [16S: EU005433; COI: EU005472; COII: EU005511; ND2: EU005538; CAD: KF214458]

FRENCH POLYNESIA: MOOREA: Three Coconuts Trail, Malaise Trap; 10.viii.2006; Claridge; 202000 [16S: EU005434; COI: EU005473; COII: EU005512; ND2: EU005539; CAD: KC692829]

HAWAIIAN ISLANDS: O’AHU: Waianae-Kaala Trail, 2300 ft.; 5.iii.2006; O’Grady, Magnacca & Bennett; 200947 [16S: EU005432; COI: EU005471; COII: EU005510; ND2: EU005536]

*Libnotes orofenaae (Alexander, 1947)*

FRENCH POLYNESIA: TAHITI: Mt Marau, 1200m, Malaise Trap; 10.v.2006; Claridge; 202034 [16S: EU005431; COI: EU005470; COII: EU005509; ND2: EU005536; CAD: KC692830]

*Geranomyia advena (Alexander, 1954)*

HAWAIIAN ISLANDS: HAWAI’I: Waipio Valley, He’elawe Falls; 12.xii.2003; O’Grady and Englund; 200104 [COI: EU005469; COII: EU005508; ND2: EU005535]

*Stryingomyia didyma Grimshaw, 1901*

FRENCH POLYNESIA: MOOREA: Three Coconuts Trail, Malaise Trap; 10.viii.2006; Claridge; 202001 [16S: EU005436; COI: EU005475; COII: EU005514; ND2: EU005541; CAD: KC692832]

**Appendix S2.** Primer names and references. Mitochondrial primer numbers correspond to the location in the *Drosophila yakuba* mitochondrial genome [1]

| **Primer name** | **Length** | **Genome** | **Ref** |
| --- | --- | --- | --- |
| Cytochrome Oxidase I (COI): 2183 or 2640 and 3041 | 824 | mitochondrial | [2] |
| Cytochrome Oxidase II (COII): 3037 and 3771 | 767 | mitochondrial | [2] |
| NADH Dehydrogenase 2 (ND2): 192 and 732 | 532 | mitochondrial | [2] |
| 16s | 534 | mitochondrial | [3] |
| sans fille (snf) | 359 | nuclear | [4] |
| rudimentary (CAD) | 864 | nuclear | [5] |

**Appendix S3.** Summary of data partitions and the nucleotide models determined for each partition, selected using BIC in PartitionFinder [6].

| **Program: loci**  **(# partitions)** | **Figure** | **Data partitions: nucleotide models** |
| --- | --- | --- |
| Mr Bayes: COI (3) | Figure S4a | position 1: GTR+G  position 2: F81+I+G  position 3: GTR+G |
| Mr Bayes: COII (3) | Figure S4b | position 1: GTR+I+G  position 2: HKY+I+G  position 3: GTR+I+G |
| Mr Bayes: ND2 (3) | Figure S4c | position 1: HKY+I+G  position 2: GTR+I+G  position 3: GTR+G |
| Mr Bayes: 16s (1) | Figure S4d | GTR+G |
| Mr Bayes: CAD (3) | Figure S4e | position 1: HKY+G  position 2: HKY+G  position 3: GTR+G |
| Mr Bayes: snf (1) | Figure S4f | HKY+I |
| Mr Bayes/RAxML: COI, COII, ND2, 16S, CAD, snf (9) | Figure 1/  Figure S5 | **Mr Bayes:**  COI, COII 1^st^ position: GTR+G  COI, COII, ND2 2^nd^ position: HKY+I+G  COI, COII, ND2 3^rd^ position: GTR+G  16s: GTR+G  ND2 1^st^ position: GTR+G  CAD 2^nd^ position: HKY+G  CAD 1^st^ position: HKY+G  CAD 3^rd^ position: GTR+G  snf: HKY+I  **RAxML:**  Each partition was run using the GTR+GAMMA model |
| BEAST: COI, COII, ND2, 16S, CAD  Island calibrations (6) | Figure 2, Figure 3,  Figure S6a | CAD, COI, COII 1^st^ position: GTR+I+G  CAD, COI, COII 2^nd^ position: HKY+I+G  COI, COII, ND2 3^rd^ position: GTR+I+G  ND2 2^nd^ position & 16s: GTR+G  ND2 1^st^ position: TrN+G  CAD 3^rd^ position: HKY+I |
| BEAST: COI, COII, ND2, 16S, CAD  COI divergence rates (7) | Figure S6b,  Figure S6c | COI: GTR+I+G  CAD, COII 1^st^ position: TrN+I+G  CAD, COII, ND2 2^nd^ position: HKY+I  COII, ND2 3^rd^ position: GTR+I+G  16s: GTR+G  ND2 1^st^ position: TrN+G  CAD 3^rd^ position: HKY+I |

**Appendix S4a.** Individual Bayesian Gene Trees – COI.


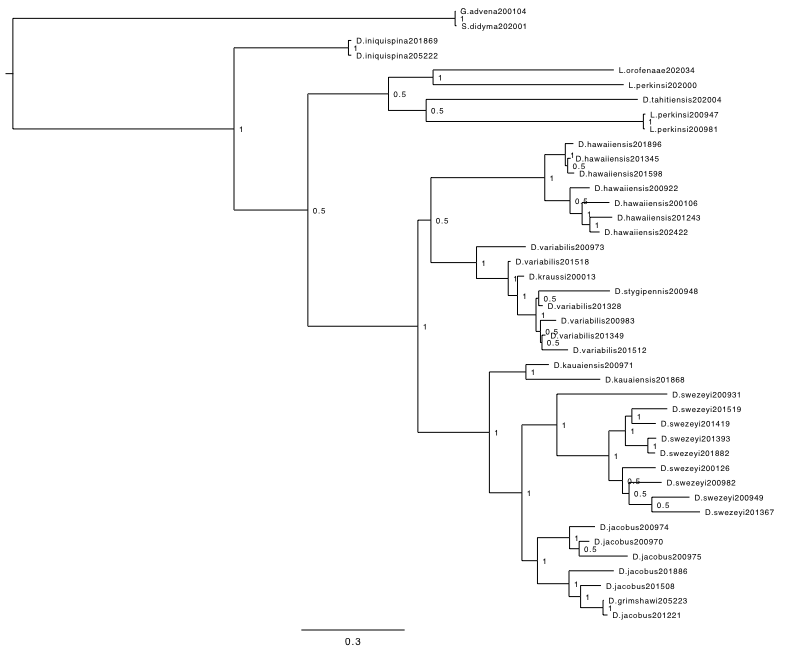


**Appendix S4b.** Individual Bayesian Gene Trees – COII.


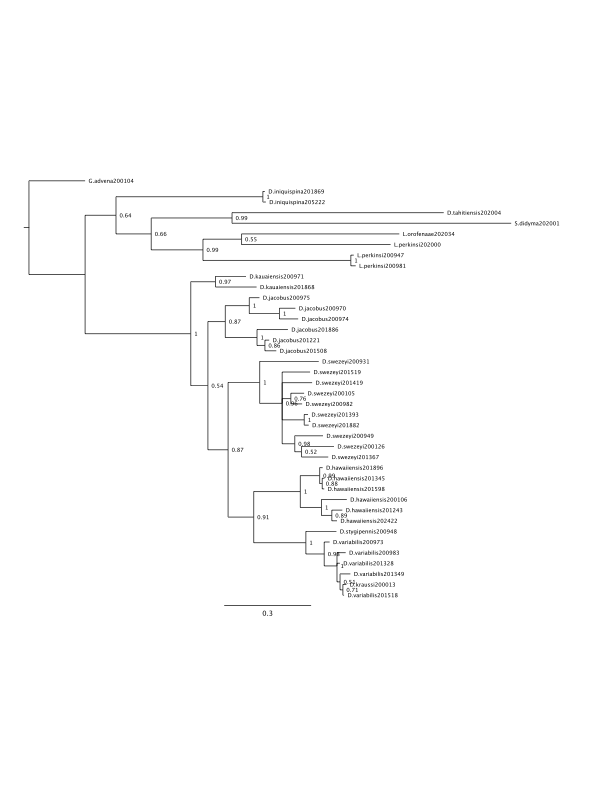


**Appendix S4c.** Individual Bayesian Gene Trees – ND2.

**
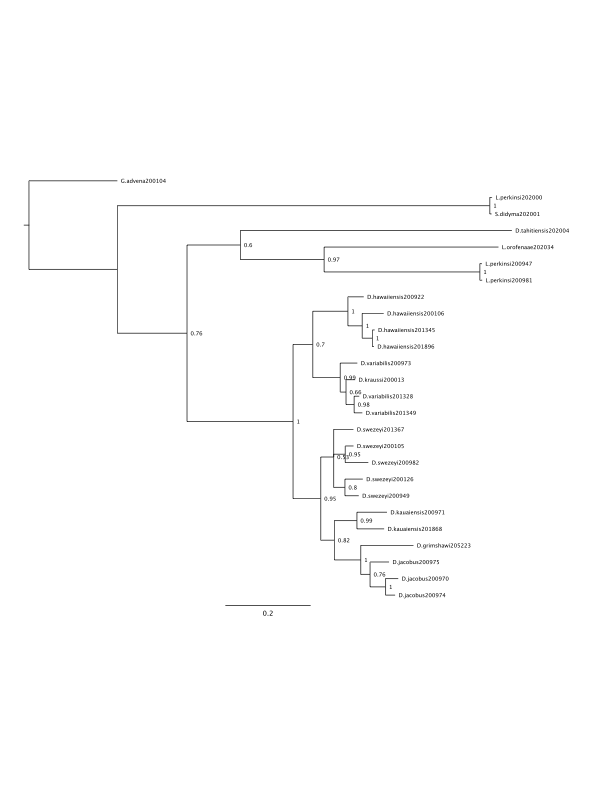
**

**Appendix S4d.** Individual Bayesian Gene Trees – 16s.


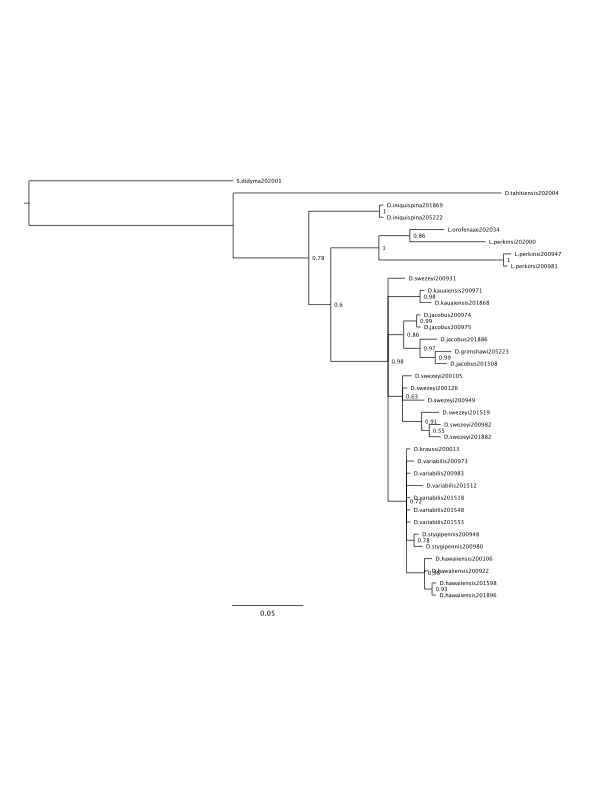


**Appendix S4e.** Individual Bayesian Gene Trees – CAD.


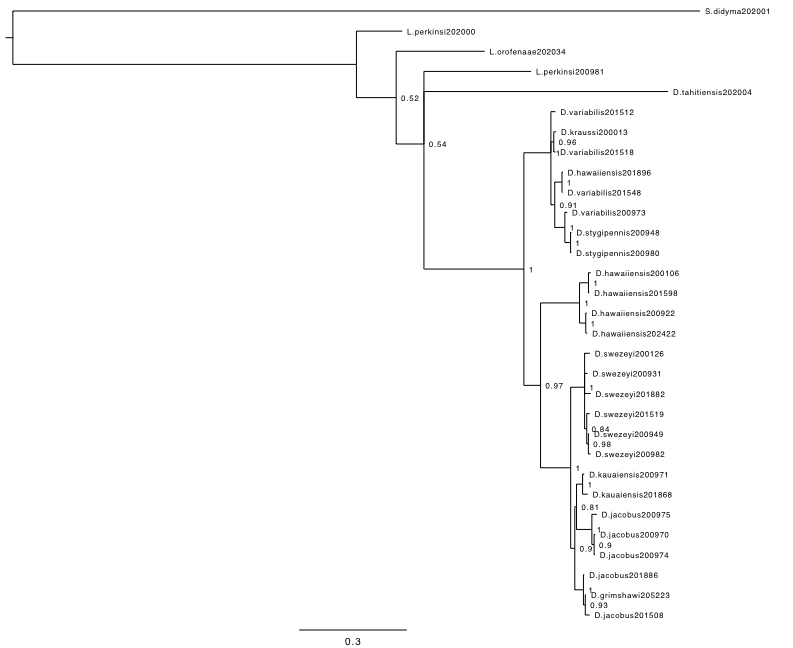


**Appendix S4f.** Individual Bayesian Gene Trees – snf.


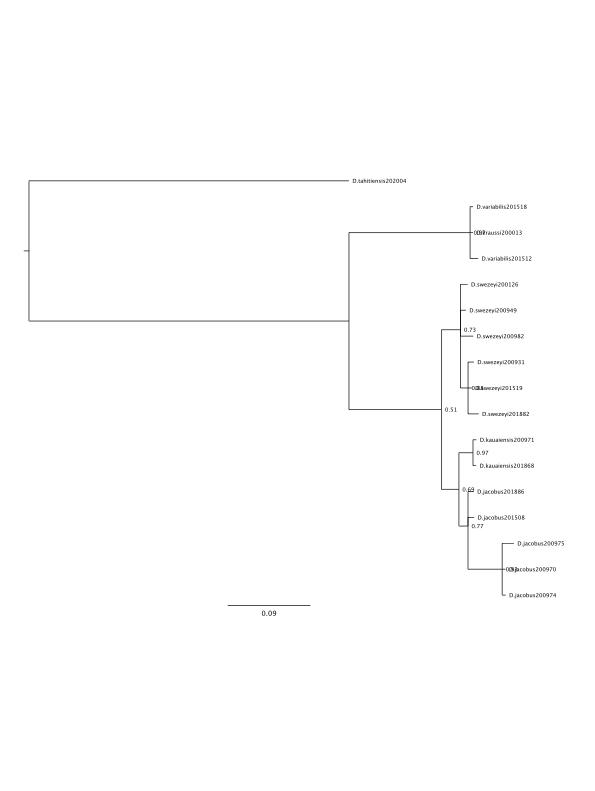


**Appendix S5.** Combined maximum likelihood analysis of all genes

**
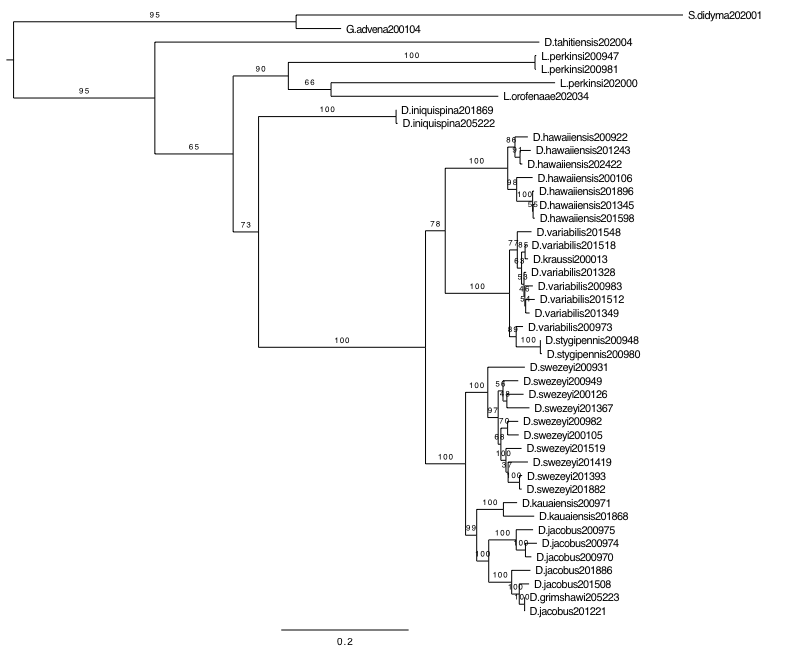
**

**Appendix S6a.** Alternate BEAST Trees: Island Calibrations I. Here, the *D. variabilis/D. kraussi* calibration is based on a Maui age and the *D. jacobus/D. grimshawi* calibration is based on a Molokai age.
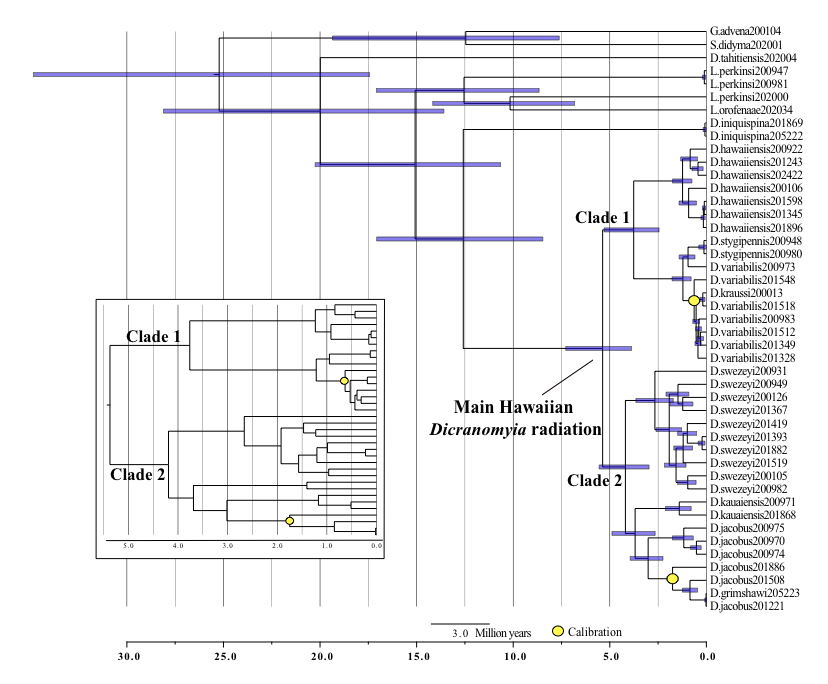


**Appendix S6b.** Alternate BEAST Trees: COI rate based on Hawaiian arthropod divergence, 5.2% sequence divergence/million years [7].

**
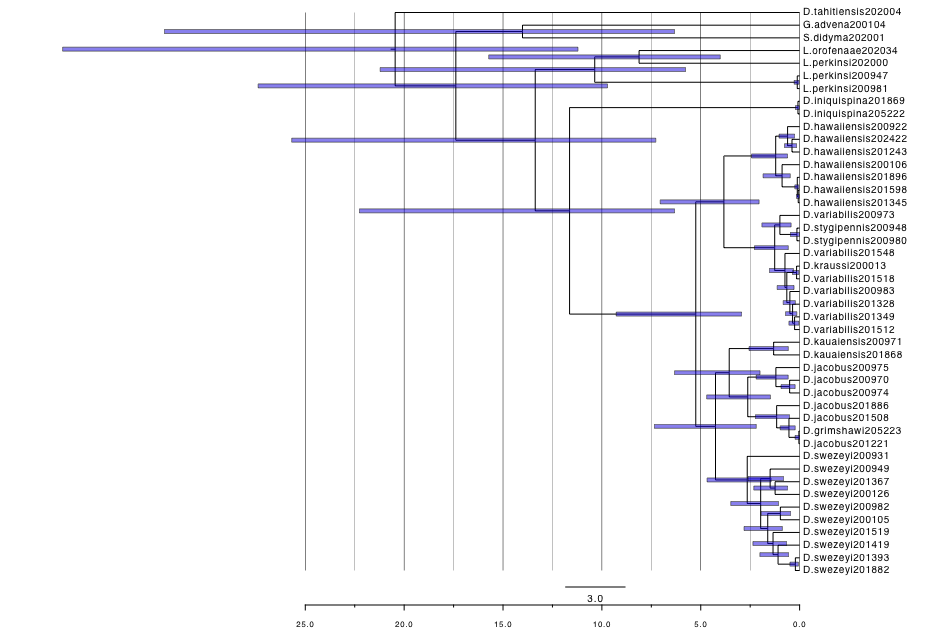
**

**Appendix S6c.** Alternate BEAST Trees: COI rate, 2.3% sequence divergence/million years [8].


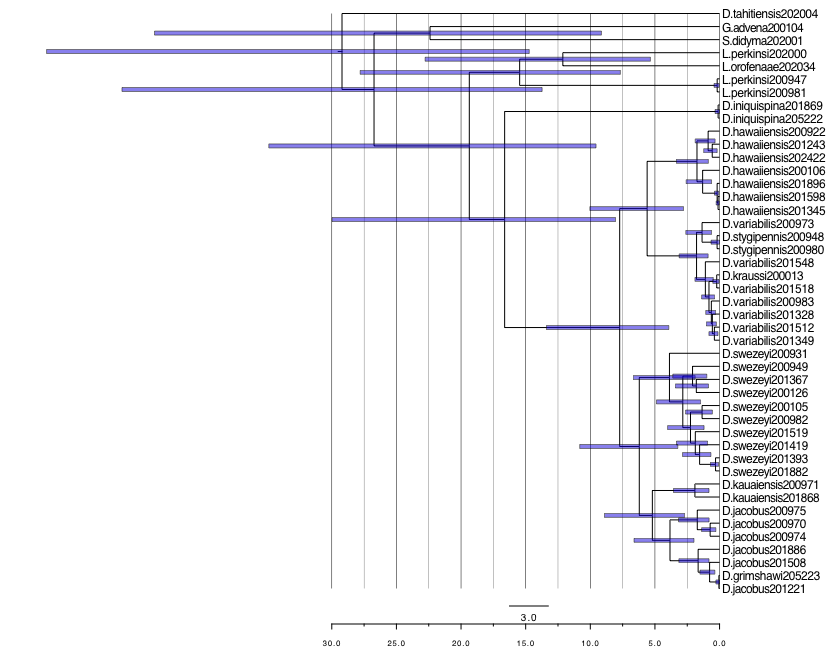


**Supplementary References Cited:**

1. Clary DO, Wolstenholme DR (1985) The mitochondrial DNA molecule of *Drosophila yakuba*: Nucleotide sequence, gene organization and genetic code. Journal of Molecular Evolution 22: 252-271.

2. Bonacum J, DeSalle R, O'Grady PM, Olivera DSCG, Wintermute J, et al. (2001) New nuclear and mitochondrial primers for systematics and comparative genomics in Drosophilidae. Drosophila Information Service 84: 201-204.

3. DeSalle R (1992) The origin and possible time of divergence of the Hawaiian Drosophilidae - evidence from DNA-sequences. Molecular Biology and Evolution 9: 905.

4. Zilversmit M, O'Grady PM, DeSalle R (2002) Shallow genomics, phylogenetics, and evolution in the family Drosophilidae. In: Altman R, Dunker AK, Hunter L, Lauderdale K, Klein T, editors. Pacific Symposium on Biocomputing.

5. Moulton JK, Weigmann BM (2004) Evolution and phylogenetic utility of cad (rudimentary) amond Mesozoic-aged Eremoneuran Diptera (Insecta). Molecular Phylogenetics and Evolution 31: 363-378.

6. Lanfear R, Calcott B, Ho SYW, Guindon S (2012) PartitionFInder: combined selection of partitioning schemes and substitution models for phylogenetic analyses. Molecular Biology and Evolution 29: 1695-1701.

7. Goodman KR, Welter SC, Roderick GK (2012) Genetic divergence id decoupled from ecological diversification in the Hawaiian *Nesosydne* planthoppers. Evolution 66: 2798-2813.

8. Brower AVZ (1994) Rapid morphological radiation and convergence among races of the butterfly Heliconius erato inferred from patterns of mitochondrial DNA evolution. Proceedings of the National Academy of Sciences 91: 6491-6495.
